# Links Between Executive Functions and Decoding Skills in a Semitransparent Orthography: A Longitudinal Study from Kindergarten to First Grade

**DOI:** 10.3390/ejihpe15020015

**Published:** 2025-01-31

**Authors:** Marisa G. Filipe, Tânia Carneiro, Sónia Frota

**Affiliations:** Center of Linguistics, School of Arts and Humanities, University of Lisbon, Alameda da Universidade, 1600-214 Lisboa, Portugal; tania.s.carneiro@edu.ulisboa.pt (T.C.); sfrota@edu.ulisboa.pt (S.F.)

**Keywords:** executive functions, word decoding, early childhood, longitudinal study

## Abstract

Despite progress in understanding the link between executive functions (EFs) (i.e., a set of skills involved in goal-directed activities crucial for regulating thoughts and actions) and word decoding skills, research has not yet determined the dynamics and extent of this link. This longitudinal study examined whether EF subcomponents (inhibition, working memory, and cognitive flexibility) significantly predict decoding skills in Portuguese, which has a semitransparent orthography. The sample included 81 children (*M*_age_ = 5.36 years, *SD*_age_ = 0.30; 40 girls) in their final year of kindergarten. EF performance was evaluated during kindergarten using nonverbal performance-based tests, and decoding skills were assessed one year later in first grade through a pseudoword reading task. A three-step regression analysis was used to explore the unique contributions of each EF subcomponent to decoding skills. Findings indicated that inhibitory control skills were significant predictors of first-grade decoding outcomes. However, adding working memory abilities to the regression model did not increase the explained variance. In the final step, including cognitive flexibility skills reduced the significance of inhibitory control and increased the amount of explained variance. These results suggest that, while inhibitory control plays an important role, cognitive flexibility has a more significant impact on word decoding skills, highlighting the importance of early development of specific EFs for decoding abilities.

## 1. Introduction

Reading is a cognitively demanding task that requires proficiency in phonological structures, accurate conversion of letter sequences into spoken language through orthographic processes, retrieval of semantic information stored in long-term memory, and integration of these elements to construct coherent text representations (Melby-Lervåg & Lervåg, 2011). Research on reading processes has highlighted the role of executive functions (EFs) (Butterfuss & Kendeou, 2018; Follmer, 2018), a set of goal-directed cognitive skills that regulate thoughts and behaviors (Miyake et al., 2000). However, the pathways through which EF subcomponents support reading acquisition still need to be explored, particularly in the context of established predictors such as word decoding and across different orthographic systems. Thus, the primary goal of this study was to explore whether particular EFs—namely inhibition, working memory, and cognitive flexibility—predict later word decoding skills in first-grade students learning a language with a semitransparent orthography.

### 1.1. Reading Acquisition

Reading acquisition requires the successful integration of multiple components, and various theories and models have been proposed to understand this complexity. The Simple View of Reading (SVR) (Gough & Tunmer, 1986) is a widely accepted theoretical model explaining reading development. According to SVR, reading comprehension results from combining decoding skills, which are essential for accurately and/or fluently translating written language into speech, with language comprehension abilities that facilitate understanding of the text (Gough & Tunmer, 1986).

Decoding skills include the cognitive process whereby written symbols, specifically graphemes (e.g., letters and combinations of letters in alphabetic writing systems), are systematically converted into corresponding spoken units (e.g., phonemes, syllables, or morphemes). This process involves sequentially integrating these units to articulate a spoken word or a nonword (Ehri, 1995, 1998). Thus, word decoding involves more than simple print-to-sound mapping; it includes a range of lexical and sublexical processes, such as phonological awareness and orthographic processing (Ehri, 2005; Goswami & Bryant, 1990).

In the initial reading acquisition stage, reading comprehension tends to rely more on word decoding than on oral language proficiency. Thus, for younger readers, mastering word decoding skills is crucial for comprehension, while oral language abilities become progressively more significant as readers become more proficient in reading (Ouellette & Beers, 2010; Storch & Whitehurst, 2002; Vellutino et al., 2007). As students develop proficient decoding skills, they can effortlessly and automatically convert letter sequences into words (Hoover & Gough, 1990).

### 1.2. Reading Acquisition and Executive Functions (EFs)

Research has increasingly underscored the critical role of EFs in reading, demonstrating their significant impact on reading development (Cirino et al., 2019; Follmer, 2018; Guajardo & Cartwright, 2016; Liu et al., 2018). EF is an umbrella term frequently conceptualized including the following core subcomponents: inhibitory control, working memory, and cognitive flexibility (also known as inhibition, updating, and shifting) (Miyake et al., 2000). Inhibition is the ability to suppress automatic responses; working memory involves storing and manipulating information during task performance; cognitive flexibility refers to switching between rules, operations, or mental states.

Although EF is an umbrella term including different subcomponents, it is often considered a unitary construct in younger children (Hughes et al., 2009; Shing et al., 2010; Tsujimoto et al., 2007; Wiebe et al., 2008). However, studies also found differentiation of EF subcomponents even in preschoolers. For example, Miller et al. (2012) observed distinct contributions of working memory and inhibition in preschool-aged children when using confirmatory factor analysis with performance indicators. Similarly, a longitudinal study by Usai et al. (2014) identified differences between working memory and shifting in children aged five and six. Therefore, the gradual differentiation of EF subcomponents remains a subject of ongoing debate, and it is crucial to understand the unique contributions of specific EF components to reading skills.

Moreover, research has highlighted the critical importance of specific EF components in reading. Butterfuss and Kendeou (2018) reviewed the roles of updating, inhibition, and shifting in this context. They emphasized the role of updating in maintaining coherent text comprehension, the function of inhibition in filtering out irrelevant information, and the potential of shifting to aid in integrating semantic and phonological information during reading, as well as in flexibly allocating attention. The authors also suggested that including other EF components, such as planning, in models of the reading process would enhance our understanding of reading development in children.

Interestingly, studies have suggested that training EFs could enhance reading abilities (Cirino et al., 2017; Dahlin, 2011), although the effectiveness of such programs remains debatable (Melby-Lervåg et al., 2016). These studies also suggested that EFs play a supportive role in children’s reading skills development.

### 1.3. Decoding Skills and EFs

Meta-analytic research has identified consistent small-to-moderate correlations between EF subcomponents and decoding skills across diverse samples, tasks, and research methodologies (Ober et al., 2020). Therefore, robust empirical evidence supports that EFs facilitate word decoding, especially during the early stages of reading development, due to the effortful nature of acquiring these abilities (Haft et al., 2019). Indeed, EFs could enhance the automatization of decoding abilities (Cartwright et al., 2019), as studies focusing on preschool-aged children have shown that EFs predict essential skills for word decoding, such as letter-word identification and phonological awareness, as well as proficiency in word decoding itself (Blair & Razza, 2007; Welsh et al., 2010).

Further studies also revealed the distinct impact of EF subcomponents, such as inhibitory control, working memory, and cognitive flexibility:

1. Inhibitory control could be crucial for decoding because readers must inhibit competing orthographic neighbors (i.e., words with similar spellings) to identify target words accurately (Massol et al., 2015). This link might be indirect, as Van de Sande et al. (2017) demonstrated that attentional and inhibitory control in kindergarten (age five) indirectly influenced reading comprehension in grade two (age seven) via phonological awareness.
2. Working memory could support decoding by allowing readers to store phonological and morphological representations while processing orthographic units incrementally. Indeed, a meta-analysis by Peng et al. (2018) found a significant correlation between decoding skills and working memory, corroborating this relationship. However, other studies found that working memory directly and indirectly impacted reading comprehension, mediated by text recall and inferencing, but not decoding ability (Ober et al., 2019).
3. Cognitive flexibility could help readers manage orthographic and phonological representations during lexical retrieval (Cartwright et al., 2017). Guajardo and Cartwright (2016) found a significant moderate association between task-switching and decoding in preschool-aged children. However, this association was no longer significant when the same children were tested four years later.

Despite the existing research on the topic, the pathways by which specific EF subcomponents influence the development of decoding abilities need to be clarified and warrant further investigation.

### 1.4. Decoding Skills and EFs: The Impact of Research Designs, EF Tasks, and Orthographic Systems

To test the hypothesis that EFs impact reading across time, longitudinal designs are essential. However, most studies linking EFs to reading are cross-sectional, failing to track reading development over time. For example, only 4 out of 29 studies reviewed by Follmer (2018) were longitudinal, and more recent research continues to highlight this limitation (Cirino et al., 2019; Liu et al., 2018; Spencer et al., 2019). Thus, longitudinal studies are necessary to understand how EFs predict reading development.

Many studies investigating the relationship between EFs and reading use language-dependent tasks, such as verbal working memory (Hjetland et al., 2018; Lervåg et al., 2017; Stipek & Valentino, 2015), that are significantly influenced by linguistic abilities. Indeed, in a meta-analysis comparing skilled and less skilled readers on EF tasks, the type of response required significantly impacted effect sizes: tasks demanding verbal responses showed higher effects than those requiring nonverbal responses (Booth et al., 2010). Thus, the EF task affects the strength of the association with decoding skills, highlighting the need for using nonverbal tasks to assess the unique contributions of EFs.

Cross-linguistic comparisons revealed that the acquisition of decoding skills varies depending on the depth of the orthographic system (Ziegler & Goswami, 2005). Orthographic depth refers to the complexity of relationships between written symbols and their corresponding sounds, influencing how predictably words are pronounced (Schmalz et al., 2015). Beginning readers encounter more significant challenges in learning to decode languages with deep orthographies, such as English, where letter-sound relationships are less consistent (Ellis et al., 2004). In contrast, languages like German, with transparent orthographies and regular phoneme-grapheme correspondences, facilitate easier decoding for early readers (Seymour et al., 2003). Thus, the deeper the orthography, the greater the demands placed on EFs. Still, research has predominantly focused on languages with opaque orthography, such as English (Berninger & Nagy, 2008), and to gain a comprehensive understanding of these relationships, further research across orthographic systems of various depths is essential.

### 1.5. Present Study

Few studies have examined the unique contribution of different EF subcomponents during kindergarten and their relation to first-grade decoding abilities. Additionally, research frequently uses tests to evaluate EFs that rely on verbal content, which can influence the results and may not fully isolate EFs from language abilities. Moreover, most studies focus on opaque languages, leaving a gap in understanding how EFs relate to reading skills in more transparent orthographies. Thus, the primary goal of this study was to explore whether specific EFs (inhibition, working memory, and cognitive flexibility) measured through nonverbal tasks in kindergarten significantly predict later decoding skills in first grade in a semitransparent orthography—European Portuguese. A semitransparent orthography will allow children to achieve fluent decoding relatively early in their school years (Seymour et al., 2003). Despite the greater consistency between letters and sounds, decoding still requires cognitive effort, as children must apply phonological decoding strategies while navigating orthographic patterns. Based on previous research, we anticipated that kindergarten children with higher performance on EF tasks would exhibit higher decoding abilities in their first grade.

Analyzing associations between inhibition, working memory, and cognitive flexibility in kindergarten and decoding skills in first grade could shed light on the developmental pathways of early literacy skills. This will support our understanding of how specific EFs contribute to acquiring and mastering reading abilities during early childhood. This knowledge could inform educational practices to support reading development and improve literacy outcomes.

## 2. Method

### 2.1. Participants

Eighty-one children in their last year of kindergarten participated in the study (*M*_age_ = 5.36 years, *SD*_age_ = 0.30; 40 girls). Convenience sampling was chosen based on the schools’ accessibility and willingness to participate. The study was conducted at two private schools in the Oporto Metropolitan Area. All participants were typically developing children and native speakers of European Portuguese. According to schools’ reports, none of the children had cognitive or language disorders. Additionally, their performance on a nonverbal intelligence test, the Raven’s Colored Progressive Matrices (Raven et al., 1995), was within the expected range for their age based on Portuguese norms adapted by Simões (2000).

### 2.2. Measures

Nonverbal intelligence. Raven’s Colored Progressive Matrices (Raven et al., 2004; Simões, 2000) were used to assess nonverbal intelligence abilities. In each item, participants were required to choose the missing element that completes a pattern from six options. The total score was calculated as the sum of correct answers, with higher scores indicating higher nonverbal intelligence abilities. This test demonstrated sensitivity to variations in intellectual functioning, showing strong test-retest reliability (*r* = 0.80) (Raven et al., 1998), as well as robust internal consistency (KR-20 and Cronbach’s alpha averaging around 0.85) (Cantwell, 1967; Simões, 1989).

Inhibitory control. The Inhibition subtest of the NEPSY-II, a Developmental Neuropsychological Assessment (Korkman et al., 2007), was employed to measure the ability to inhibit automatic responses in favor of new ones. Participants were presented with one sheet featuring black-and-white shapes (squares and circles) and another with arrows (pointing up and down). They were instructed to verbally respond with the opposite shape (e.g., saying “square” for “circle” and vice versa) or arrow direction (e.g., saying “up” for “down” and vice versa). The combined inhibition score that included both completion time and errors was used in the analyses. This score was derived by integrating the percentile rank for the total of errors and the scaled score for the total completion time (retrieved from the relevant tables in the Clinical and Interpretation Manual). A higher combined score indicates higher inhibition skills. The Inhibition subtest has shown good test-retest reliability (*r* = 0.81) (Brooks et al., 2010) and excellent internal consistency (Cronbach’s alpha = 0.92) (Korkman et al., 2007).

Working memory. We assessed nonverbal working memory through the Corsi Blocks task of the Coimbra Neuropsychological Assessment Battery (Simões et al., 2016), one of the most common tests used to measure visuospatial short-term and working memory (Corsi, 1972). In this task, the examiner taps on a board with nine blocks in predetermined sequences, and the child must reproduce each tapping pattern. Each item consists of two trials containing the same number of blocks but utilizing different sequences. The final score was the number of sequences successfully recalled, with higher scores indicating higher nonverbal working memory skills. The temporal stability coefficients of the results were *r* = 0.60 (Moura et al., 2018).

Cognitive flexibility. Participants were evaluated with the Children’s Color Trails Test (CCTT) (Llorente et al., 2003). The CCTT was developed to evaluate aspects similar to those of the Trail Making Test while minimizing the influence of linguistic components. The CCTT consists of two parts: CCTT-1 and CCTT-2. In both parts of the test, participants connect circled numbers (1–15) using a pencil in ascending order. In the CCTT-2, however, the numbers must alternate in color, specifically pink and yellow. CCTT-1 measures visual tracking, processing speed, and graphomotor skills. CCTT-2 adds divided attention, set-switching, inhibition, and working memory/sequencing. The task-switching demands of CCTT-2 reflect a core aspect of cognitive flexibility, where individuals have to rapidly change from one task to another. The time in seconds to complete the CCTT-2 task was considered the outcome measure, and higher scores indicate lower cognitive flexibility skills. This task exhibited good temporal stability, with coefficients ranging from 0.90 to 0.99 across different time intervals. The CCTT demonstrates a strong level of convergent validity compared to other instruments designed to assess EFs.

Decoding skills. The ALEPE—Pseudoword Reading subscale (Sucena & Castro, 2011) was used to assess decoding abilities. The child should read a list of pseudowords in isolation, which includes 4 practice items and 15 test items. The stimuli had different orthographic complexity: simple and complex pseudowords. The primary outcome measure was the number of correct responses. Higher scores are associated with higher decoding skills. The internal consistency assessed by Cronbach’s alphas ranged between 0.46 for first graders and 0.72 for second, third, and fourth graders.

### 2.3. Procedure

All children were assessed in two 45-min individual sessions during the first term of the academic year (September–December): one session to assess EFs (Inhibition subtest of the NEPSY-II, Corsi Blocks, and CCTT) and the other to assess nonverbal intelligence and decoding abilities (Raven’s Colored Progressive Matrices and Pseudowords Reading). Additional tasks were administered but are not detailed in this report as they fall outside the scope of the present study. Participants were reassessed during their first grade, one year later. An attrition rate of 4.9% was observed between the two time points. All assessments were conducted in a quiet environment by trained research assistants with a degree in psychology.

Participant recruitment adhered to the ethical principles and guidelines outlined by the European Union Agency for Fundamental Rights and the Declaration of Helsinki (established by the World Medical Association). The study was approved by the Ethics Committee of the author’s university, and informed consent was obtained from the legal guardians of all participants, along with child assent.

### 2.4. Statistical Analyses

The independent variables included: (i) inhibition—assessed through the Inhibition subtest, which measures the ability to inhibit automatic responses; (ii) working memory—measured using the Corsi Blocks, an indicator of spatial and visuospatial working memory; and (iii) cognitive flexibility—evaluated by the CCTT-2, a test that assesses the ability to shift cognitive strategies and flexibly adapt to changing task demands. The dependent variable was decoding, measured by the performance in a pseudowords reading task. 

First, correlations between all variables explored the relationship between nonverbal intelligence, EF subcomponents (inhibitory control, working memory, and cognitive flexibility), and decoding abilities. Afterward, hierarchical regression models were employed to identify the unique and additional contribution of the EFs to decoding outcomes. As several studies have suggested a gradual emergence of EFs, beginning with inhibitory control, advancing to improvements in working memory, and subsequently to cognitive flexibility (Diamond, 2013; Miller et al., 2012; Monette et al., 2015; Usai et al., 2014), the variables were sequentially entered into the model across three steps: Step 1 included inhibitory control, Step 2 added working memory, and Step 3 introduced cognitive flexibility. The incremental R^2^ (ΔR^2^) values and associated *t*-tests statistically assessed these specific contributions.

## 3. Results

Table 1 presents descriptive statistics for all variables. The skewness values from all variables were below |1.5|, and kurtosis values were below |2.5|, indicating no significant departures from normal distribution (Kline, 2005). Although linear regression is robust to minor departures from normality, we report these values to assess potential severe deviations that could affect parameter estimates. Residuals did not significantly deviate from a normal distribution (Shapiro–Wilk: W = 0.98, *p* = 0.17). The inspection of the correlation matrices (see Table 1) revealed that the performance on tasks assessing different EF subcomponents was correlated with each other. Additionally, better performance in inhibitory control, working memory, and cognitive flexibility were correlated with higher performance in the task-assessing decoding skills.

Step 1 of the regression analysis included inhibitory control as the predictor of decoding abilities. This model was significant (*R*² = 0.06, *F*(1, 69) = 4.38, *p* = 0.040). Inhibitory control (*b* = 0.35, *β* = 0.24, *t* = 2.09, *p* = 0.040) significantly predicted decoding performance.

Step 2 added short-term/working memory as a predictor of decoding abilities. Including this variable did not significantly increase the explained variance (ΔR² = 0.01, F_change_(1, 68) = 2.52, *p* = 0.089). In this model, neither inhibitory control (*b* = 0.31, *β* = 0.22, *t* = 1.79, *p* = 0.078) nor working memory (*b* = 0.23, *β* = 0.10, *t* = 0.83, *p* = 0.411) were significant predictors.

Step 3 introduced cognitive flexibility to the model, significantly increasing the amount of explained variance (ΔR² = 0.12, F_change_(1, 67) = 5.18, *p* = 0.003). Cognitive flexibility (*b* = −0.01, *β* = −0.38, *t* = −3.14, *p* = 0.003) emerged as a significant and unique predictor. When cognitive flexibility was included, both inhibitory control (*b* = 0.24, *β* = 0.17, *t* = 1.43, *p* = 0.157) and working memory (*b* = −0.10, *β* = −0.04, *t* = −0.349, *p* = 0.728) remained non-significant, indicating that lower cognitive flexibility (i.e., higher completion time) is associated with poorer reading performance.

The detailed parameter estimates for the regression models are presented in Table 2. The inspection of the variance inflation factor (VIF) showed no evidence of multicollinearity (VIF < 1.3 for all predictors), confirming that the relationships between predictors do not substantially affect the variance of the estimated coefficients. Durbin–Watson equals 1.684, indicating no substantial autocorrelation in the residuals.

## 4. Discussion

This longitudinal study explored the link between EF performance during kindergarten and later decoding skills. Our findings provided valuable insights into the specific contributions of inhibitory control, working memory, and cognitive flexibility, measured through nonverbal tasks, to developing decoding abilities in a semitransparent orthography. Results showed significant correlations between all EF subcomponents, highlighting their expected interconnected nature (Diamond, 2013). The three-step regression analysis revealed that inhibitory control initially predicted decoding outcomes, but including working memory in Step 2 did not increase the explained variance. In Step 3, the significance of inhibitory control disappeared when cognitive flexibility was introduced into the model, significantly increasing the amount of explained variance. Overall, the results align with the meta-analysis findings from Ober et al. (2020), indicating that aspects of EFs, especially task-switching and inhibitory control, contribute uniquely to decoding abilities. Thus, our findings underscore the importance of inhibitory control and especially cognitive flexibility in reading acquisition, even in kindergarten, suggesting that specific EFs play a more significant role than previously recognized.

Our results support the idea that EFs may play a pivotal role in the early stages of reading development due to their effortful nature (Haft et al., 2019) and could enhance the automatization of the reading process (Cartwright et al., 2019), highlighting the significant influence of cognitive flexibility in managing the complex demands of early reading tasks and supporting the development of efficient decoding skills. The prominent unique role of cognitive flexibility supports the hypothesis that this particular EF component is important for handling reading tasks that are dynamic and complex (Cartwright et al., 2017; Ober et al., 2019). Indeed, cognitive flexibility emerged as the most significant predictor of decoding performance in early reading development. The negative association—where higher completion time in the cognitive flexibility task is linked to poorer performance in the decoding task—suggests that children who struggle with cognitive flexibility may find it challenging to manage orthographic and phonological representations effectively, thereby hindering their decoding abilities.

The significant role of inhibitory control in this study also aligns with meta-analytic evidence of its influence on decoding skills (Ober et al., 2020). Results suggest that inhibitory control enables young readers to suppress irrelevant information, such as competing orthographic neighbors, and focus on decoding words, along the lines of Massol et al. (2015). However, the significance of inhibitory control diminished when cognitive flexibility was introduced into the model, indicating that cognitive flexibility has a more substantial impact on decoding abilities.

The non-significant influence of working memory on decoding skills observed in our study contrasts with findings from prior research (Welsh et al., 2010). Several factors may explain this disparity. First, variations in the domains of working memory assessed (e.g., verbal vs. non-verbal) could influence the observed effects on decoding abilities. Second, differences in participant age across studies might contribute to varying results as the development and utilization of working memory capacities vary with age. Finally, the type of tasks used to measure decoding skills could also play a role, aligning with findings from Peng et al. (2018) meta-analysis that highlighted stronger associations between working memory and word recognition than nonword reading. Furthermore, existing models already emphasize their significance for more complex reading abilities, such as reading comprehension (Baddeley et al., 1985; Cain et al., 2004; Daneman & Carpenter, 1980; Engle et al., 1992; Ericsson & Kintsch, 1995), suggesting a reduced impact on decoding skills from a theoretical standpoint. These considerations underscore the complexity and context-dependent nature of working memory’s role in early reading development.

Overall, the present findings support the differentiation of EFs into distinct components, even in kindergarten-aged children. In contrast to the view that EF subcomponents are uniformly integrated into a single construct in younger children (Hughes et al., 2009; Shing et al., 2010; Tsujimoto et al., 2007; Wiebe et al., 2008; Wiebe et al., 2011), our study highlights the unique developmental trajectories of specific EF components during early childhood. Previous research consistently identifies two primary EF domains in this age group—working memory and inhibitory control (Karr et al., 2018; Lerner & Lonigan, 2014; St. Clair-Thompson & Gathercole, 2006)—while our findings further highlight the critical role of cognitive flexibility. This aligns with studies demonstrating significant developmental changes in cognitive flexibility early in development (Blaye et al., 2006; Crone et al., 2004; Dick, 2014; Filipe et al., 2023; Magalhães et al., 2020; Rosselli & Ardila, 1993; Welsh et al., 1991). Thus, our results contribute to the research on EFs by suggesting distinct developmental trajectories for these components in early childhood rather than viewing EFs as a unified construct.

The longitudinal nature of this study allowed the examination of the developmental trajectories of EFs and decoding abilities over time, providing more robust evidence of the predictive relationships than cross-sectional designs. However, the study has limitations that warrant further investigation. The relatively small sample size implies caution when generalizing these findings. Furthermore, the study’s focus on a semitransparent orthography may only partially capture the variability in EF contributions across languages with different orthographic depths. The low internal consistency of the pseudoword decoding task should also be considered, as the relatively small number of stimuli could lead to significant variability in the assessment of decoding abilities. Future research should explore ways to improve the reliability of decoding assessments (e.g., increasing the number of stimuli or using alternative methods to assess word decoding). Additionally, future studies should consider larger sample sizes and diverse orthographic contexts to explore variations in the relationships between EFs and decoding skills. Moreover, longitudinal studies across languages with different orthographic depths will enhance our understanding of these dynamics and inform more effective interventions.

These findings could have practical implications for educational practices to promote reading skills. Given the significant role of inhibitory control and, in particular, cognitive flexibility, targeted interventions to enhance these EF components could bolster decoding abilities in early readers. Scaffolding EF skills in kindergarten can ultimately support reading comprehension by boosting word decoding skills. Despite the methodological limitations, EF interventions have shown promising effects in young children (Diamond & Lee, 2011; Röthlisberger et al., 2012; Veloso et al., 2020). Furthermore, EFs can be used as additional indicators of early reading difficulties, which may help to prevent widening achievement gaps in reading over the school years. However, these implications warrant further investigation, as while interventions targeting inhibitory control and cognitive flexibility show promise for enhancing decoding abilities in early readers, robust studies to substantiate these findings are needed.

In conclusion, the present longitudinal study adds to the robust empirical evidence supporting the role of EFs in facilitating word decoding, which is particularly crucial during the early stages of reading development (Haft et al., 2019). While inhibitory control is important, cognitive flexibility appears to play a more significant role in decoding within semitransparent orthographies, underscoring its importance in managing multiple cognitive demands and integrating orthographic and phonological information during reading. The findings suggest that interventions aiming to enhance reading skills in young children should consider targeting cognitive flexibility alongside traditional decoding strategies. The significant roles of inhibitory control and cognitive flexibility highlight the importance of these cognitive skills in supporting young readers’ ability to decode words, offering valuable directions for both future research and educational interventions.

## Figures and Tables

**Table 1:** Descriptive statistics (mean and standard deviation) and bivariate correlations between measures.

	Descriptive Statistics		Bivariate Correlations			
Variables	*M*	*SD*	1	2	3	4
1. Nonverbal intelligence	18.31	4.39				
2. Decoding	7.64	3.46	0.089			
3. Inhibitory control	9.43	2.52	0.33 **	0.23 *		
4. Short-term/Working memory	6.67	1.87	0.36 **	0.38 **	0.25 *	
5. Cognitive flexibility	224.46	114.28	−0.24 *	−0.40 **	−0.24 *	−0.30 *

* *p* < 0.05. ** *p* ≤ 0.01.

**Table 2:** Parameter estimates for the regression models predicting decoding abilities.

Predictors		*B*	SE	β	*t*
Step 1					
	Inhibitory control	0.35	0.17	0.24	2.09 *
Step 2					
	Inhibitory control	0.31	0.18	0.22	1.79
	Short-term/Working memory	0.23	0.27	0.10	0.83
Step 3					
	Inhibitory control	0.24	0.17	0.17	1.43
	Short-term/Working memory	−0.10	0.28	−0.04	−0.35
	Cognitive flexibility	−0.01	0.00	−0.38	−3.14 *

* p < 0.05.

## Data Availability

Given the restrictions imposed by the Ethics Committee, the detailed data from this study are available from the corresponding author, M.F., upon request.
